# Association between dietary intake of vitamin D and risk of depression, anxiety, and sleep disorders among physically active adults: a cross-sectional study

**DOI:** 10.3389/fnut.2024.1339152

**Published:** 2024-02-08

**Authors:** Vajiheh Arabshahi, Mehrad Khoddami, Maryam Milajerdi, Mahdi Moabedi, Alireza Milajerdi

**Affiliations:** ^1^Department of Nutrition, School of Public Health, Iran University of Medical Sciences, Tehran, Iran; ^2^Institute for Basic Sciences, Research Center for Biochemistry and Nutrition in Metabolic Diseases, Kashan University of Medical Sciences, Kashan, Iran; ^3^Department of Medical Sciences, Islamic Azad University, Kashan, Iran; ^4^Student Research Committee, Kashan University of Medical Sciences, Kashan, Iran

**Keywords:** vitamin D, depression, anxiety, sleep, Iran

## Abstract

**Introduction:**

Rare studies have been done to investigate the association between dietary intakes of vitamin D and the risk of mental health disorders among athletes. The current study aimed to investigate the association between this vitamin intake and the risk of depression, anxiety, and sleep disorders among a group of Iranian physically active adults.

**Methods:**

This cross-sectional study was conducted among 690 healthy athletes (18–50 years, mean BMI between 20 and 30) in Kashan, Iran. The usual dietary intake of participants was assessed by a 147-item FFQ. Depression was assessed by the Beck Depression Inventory-II (21-item), anxiety by the Beck Anxiety Inventory (21-item), and sleep disorders by the Pittsburgh Sleep Quality Index questionnaires. Statistical analyses were done by using SPSS version 18. *p* values < 0.05 were considered significant.

**Results:**

No significant association was found between vitamin D dietary intake and risk of depression in the full-adjusted model (OR: 0.96, 95% CI: 0.62, 1.51). In contrast, participants at the highest tertile of vitamin D consumption had a 49% lower risk of anxiety than those at the lowest tertile (OR: 0.51, 95%: 0.29, 0.87). Moreover, a significant 46% lower risk of sleep disorders was found among those with the highest intake of vitamin D in comparison to participants with the lowest intake (OR: 0.54, 95% CI: 0.37, 0.78).

**Conclusion:**

We found a significant association between dietary vitamin D intake and reduced risk of anxiety and sleep disorders, but not with depression, in this study. Further prospective studies are recommended for future investigations.

## Introduction

Today, the prevalence of mental disorders has increased worldwide (1). Depression and anxiety are the most common mental disorders that put a great burden on public health systems (2). Antidepressants and psychotherapy are effective in improving symptoms of depression. However, more than half of patients who used these medications reported side effects (3). It is estimated that 350 million people around the world (4), and around 40% in Iran are suffering from depression and anxiety (5).

Oxidative stress and changes in usual dietary intakes put athletes at risk for some mental disorders along with sleep disturbances (6). Continuous high-intense exercise increases the amounts of pro-inflammatory cytokines in the blood (7). In addition, athletes might change their usual dietary intake to improve their performance (8). Some of these unhealthy changes expose them to psychological distress (9, 10).

Exercise has a role in regulating sleep. Some neurotransmitters are released during exercise including dopamine, norepinephrine, serotonin, and acetylcholine which may stabilize and improve mood and decrease the risk of depression and anxiety (11). Exercise also prevents shrinkage of the hippocampal brain region which has been observed in depressed rodents, protects your brain from age-related neurodegeneration and associative diseases including cognitive decline (12), supports recovery from brain injury (such as a traumatic brain injury and even stroke) (13) and increases levels of BDNF (brain-derived neurotrophic factor) has wider implications for boosting cognition, thinking skills, memory and learning (14). Exercise can also lower depression scales (15).

Vitamin D is a fat-soluble vitamin that is synthesized by direct exposure to sunlight. A 15–20 min direct exposure to the sun can supply adequate vitamin D. However, considering cloudy days, nearly 90% of the people of the United Kingdom may have Vitamin D deficiency during the winter and spring, and 60% have insufficient concentrations (16). Vitamin D (Vit D) deficiency is an important public health concern in many populations (17). Vit D deficiency increases inflammation in the body (18, 19). Several studies have linked vitamin D deficiency to the increased risk of psychological disorders, which might be partially due to the increased inflammatory processes in the body (20, 21). A cross-sectional study found that increasing the dietary intake of vitamin D significantly reduced the risk of mental anxiety and menstrual irregularities in relatively young endurance athletes (22). A retro-prospective study found a significant association between vitamin D status and the risk of depression among military athletes (23). Some other studies revealed that vitamin D supplementation significantly reduced the incidence of psychological disorders in animal studies (24). A meta-analysis by Spedding et al. (25) states that ≥800 I.U. daily of vitamin D supplementation would be favorable for depressed individuals.

Due to a lack of relevant human studies on the association of Vit D dietary intake with the risk of mental disorders in physically active persons and the importance of such diseases in athletes, we aimed to do this cross-sectional study about the association between daily intakes of Vit D and risk of depression, anxiety, and sleep disorders in a group of Iranian physically active adults. As far as we can tell, this is the first study on the association of dietary vitamin D intake with the risk of depression, anxiety, and sleep disorders among physically active adults.

## Methods

### Participants

This cross-sectional study was conducted among 690 athletes in Kashan, Iran. Participants were selected from ten body-building clubs, 1 woman’s and 1 men’s club in each municipality region (Kashan has 5 regions). The participants were selected using the simple sampling method when they met the inclusion criteria. Healthy adults (18–50 years), mean BMI between 20 and 30, who exercised at least 3 days per week, each session lasted at least 1 h, were included. We excluded individuals who were taking vitamin D supplements. A written consent form was completed for all participants. This study has been approved by the Ethics Committee of Kashan University of Medical Sciences (IR.KAUMS.MEDNT.REC.1402.082).

### Assessments

All the participants completed a general information questionnaire consisting of questions about demographic information, and history of diseases and medications, the Beck Depression Questionnaire, Beck Anxiety Questionnaire, and Pittsburgh Sleep Quality Questionnaire. Participants’ dietary intake was evaluated by a validated food frequency questionnaire (FFQ).

### Assessment of vitamin D intake

The usual dietary intake of participants was assessed by a 147-item FFQ and its validity and reliability have been reported previously (26). The frequency of each food consumption is asked on a daily, weekly, monthly, or yearly basis. The energy and nutrient contents of each food, including amounts of vitamin D, were obtained from the US Department of Agriculture’s (USDA) national nutrient tables (27). We calculated daily intakes of all foods and dishes and converted them to grams per day using the booklet of “household measures.” Daily intake of micronutrients and macronutrients was calculated using the Nutritionist IV software modified for Iranian foods.

### Outcome assessment

Depression was assessed by the Beck Depression Inventory-II (BDI-II, 21-item) (28). Beck’s Depression Questionnaire contains 21 questions to measure the feedback and symptoms of depressed patients. Participants must answer questions on a four-choice scale ranging from zero to three. This questionnaire contains 2 questions about emotional issues, 11 questions related to cognitive issues, 2 questions related to behavior, 5 questions related to physical symptoms, and 1 question related to interpersonal behaviors. The total score of each person will be calculated, which ranges from a minimum of 0 to a maximum of 63. Patients with a depression score of higher than 10 were considered to have moderate to severe depression.

The anxiety score was calculated by the Beck Anxiety Inventory (BAI, 21-item) (29). This questionnaire is a scale with 21 questions in which participants choose one of four options to indicate the intensity of anxiety (with a score ranging from 0 to 3). Each question describes one of the common symptoms of anxiety (mental, physical, and panic symptoms). The total score of this questionnaire ranges from 0 to 63. Patients with anxiety scores of higher than 8 were considered to have moderate to severe anxiety.

The Pittsburgh Sleep Quality Index (PSQI) questionnaire was used to evaluate the sleep quality of participants (30). This questionnaire has 7 scales, which are about the mental quality of sleep, delay in falling asleep, length of useful sleep, sleep adequacy, sleep disorders (waking up at night), amount of hypnotic drug use, and disturbances in daily functioning (problems caused by insomnia during the day). Each question has a score between 0 and 3. A score of 3 on each scale indicates the maximum negative. The total score of this questionnaire is ranged from 0 to 21. A total score of 6 or above indicates inappropriate sleep quality.

### Statistical analysis

Participants were categorized into tertiles based on dietary intake of Vit D. General characteristics and age, sex, and energy-adjusted dietary intakes of participants across tertiles of Vit D were compared using one-way ANOVA for continues variables and chi-square for categorical variables. The association of vitamin D intake with the risk of depression, anxiety, and sleep disorders was assessed by using logistic regression in different models. Age (continues) and energy intake (Kcal/d) were adjusted for in the first model. Additional controlling for gender (male/female), marital status (yes/no), and smoking (yes/no) were done in the second model. Further adjustment was done for BMI (continues) in the third model. Statistical analyses were done by using SPSS version 18. *p* values < 0.05 were considered significant.

## Results

**Figure 1:**
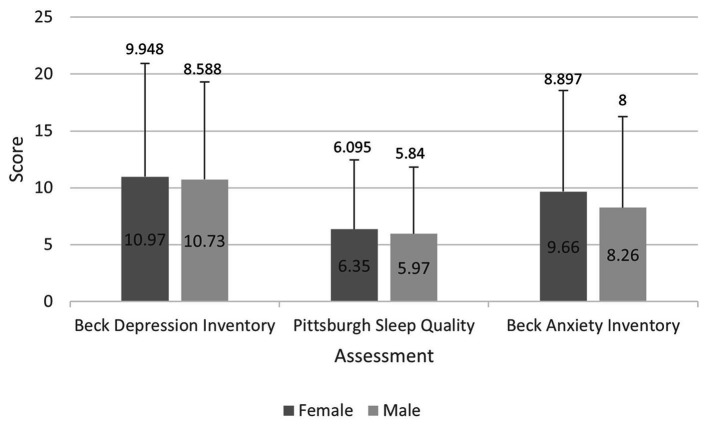
Mean and standard deviations of depression, anxiety, and sleep scores in men and women enrolled in this study.

Overall, 690 individuals enrolled in this study. Mean scores for depression in women and men were 10.97 ± 9.948 and 10.97 ± 8.588, respectively (Figure 1). General characteristics of the study population across tertiles of dietary Vit D intakes have been reported in Table 1. No significant differences were seen in mean age and BMI between tertiles of Vit D intake. The percentage of university-graduated participants had no significant differences between tertiles of vitamin D intake (*p* = 0.48). However, we found significant differences in the distribution of females as well as in married and employed participants’ distribution between tertiles of Vit D intake (*p* < 0.01).

**Table 1:** General characteristics of participants across tertiles of dietary vitamin D intake.

Variables	Vitamin D intake			*p* ^a^
	T_1_ (*n* = 230)	T_2_ (*n* = 230)	T_3_ (*n* = 230)	
Age (years)	30.45 ± 8.67	28.86 ± 9.97	28.96 ± 9.01	0.12
BMI (kg/m^2^)	24.46 ± 3.89	23.83 ± 3.56	23.68 ± 4.10	0.07
Female (%)	77.0	62.6	70.9	<0.01
Married (%)	56.5	41.7	39.1	<0.01
University graduated (%)	67.8	63.5	66.5	0.48
Employed (%)	19.6	17.8	16.5	<0.01

Data are means ± standard deviations unless indicated.

a Obtained using one-way ANOVA for continuous variables and Chi-square test for categorical variable.

Table 2 shows dietary intakes of micro- and macro-nutrients through the tertiles of Vit D consumption. Significant differences were seen in mean energy and macro-nutrient intake through the tertiles of Vit D consumption. However, dietary intakes of Iron (*p* = 0.89), magnesium (*p* = 0.72), and caffeine (*p* = 0.17) were significantly different between those categories.

**Table 2:** Dietary intakes of micro- and macro-nutrients across tertiles of vitamin D intake.

Variables	Vitamin D intake			*p* ^a^
	T_1_ (*n* = 230)	T_2_ (*n* = 230)	T_3_ (*n* = 230)	
Energy (kcal)	2950.44 ± 1576.66	2862.73 ± 771.41	2237.17 ± 1191.07	<0.001
Protein (g)	102.98 ± 51.79	113.94 ± 25.51	106.29 ± 26.35	<0.01
Carbohydrate (g)	543.92 ± 218.24	509.22 ± 76.65	497.49 ± 59.89	<0.01
Total fat (g)	137.45 ± 107.72	147.68 ± 32.14	157.24 ± 30.75	<0.01
Magnesium (mg)	467.32 ± 265.89	454.49 ± 116.51	456.29 ± 133.14	0.72
Iron (mg)	29.17 ± 20.25	29.57 ± 6.45	28.99 ± 8.02	0.89
Caffeine (mg)	58.05 ± 83.15	58.53 ± 81.98	74.06 ± 137.71	0.17

Data are means ± SD.

a Obtained using one-way ANCOVA, adjusted for age, sex and energy intake.

The odds ratio and 95% CI of depression, anxiety, and sleep disorders across tertiles of vitamin D intake have been shown in Table 3. No significant association was found between vitamin D dietary intake and risk of depression in the crude model (Odds Ratio (OR): 1.02, 95% CI: 0.66, 1.58). This finding remained unchanged in different models of confounder adjustment and in the full-adjusted model (OR: 0.96, 95% CI: 0.62, 1.51).

**Table 3:** Odds of depression, anxiety, and sleep disorders across tertiles of vitamin D intake.

Variables	Vitamin D intake			*p*-trend
	T_1_ (*n* = 230)	T_2_ (*n* = 230)	T_3_ (*n* = 230)	
Depression				
Crude	1.00 (ref.)	0.85 (0.55, 1.29)	1.02 (0.66, 1.58)	0.93
Model 1	1.00 (ref.)	0.83 (0.54, 1.26)	1.00 (0.64, 1.53)	0.97
Model 2	1.00 (ref.)	0.78 (0.51, 1.20)	0.96 (0.62, 1.49)	0.84
Model 3	1.00 (ref.)	0.78 (0.50, 1.20)	0.96 (0.62, 1.51)	0.86
Anxiety				
Crude	1.00 (ref.)	0.51 (0.30, 0.87)	0.54 (0.31, 0.92)	0.03
Model 1	1.00 (ref.)	0.49 (0.29, 0.84)	0.52 (0.30, 0.90)	0.02
Model 2	1.00 (ref.)	0.46 (0.27, 0.80)	0.51 (0.29, 0.87)	0.01
Model 3	1.00 (ref.)	0.46 (0.27, 0.80)	0.51 (0.29, 0.87)	0.01
Sleep disorders				
Crude	1.00 (ref.)	0.58 (0.40, 0.84)	0.55 (0.38, 0.80)	<0.01
Model 1	1.00 (ref.)	0.56 (0.38, 0.81)	0.53 (0.36, 0.78)	<0.01
Model 2	1.00 (ref.)	0.56 (0.38, 0.82)	0.54 (0.37, 0.80)	<0.01
Model 3	1.00 (ref.)	0.55 (0.37, 0.81)	0.54 (0.37, 0.78)	<0.01

*p*-values obtained from binary logistic regression. Model 1: adjusted for Age (continues) and energy intake (Kcal/d). Model 2: additionally adjusted for gender (male/female), marital status (yes/no), and smoking (yes/no). Model 3: further adjusted for BM (continues).

With regards to the risk of anxiety, a higher intake of vitamin D was associated with a reduced risk of this psychological disease before any adjustment (OR: 0.54, 95% CI: 0.31, 0.92). This finding was also reached in different models as well as in the final model, such that those at the highest tertile of vitamin D consumption had a 49% lower risk of anxiety than those at the lowest one (OR: 0.51, 95%: 0.29, 0.87).

Participants at the highest tertile of vitamin D consumption had a lower risk of sleep disorders than those at the bottom tertile (OR: 0.55, 95% CI: 0.38, 0.80). Full-adjusted model of analysis showed a significant 46% lower risk of sleep disorders among those with the highest intake of vitamin D in comparison to participants with the lowest intake (OR: 0.54, 95% CI: 0.37, 0.78).

## Discussion

The current study showed a significant association between dietary vitamin D intake and reduced risk of anxiety and sleep disorders among physically active adults. However, no significant association was found between vitamin D intake and the risk of depression among those participants.

Our study showed that the highest intake of vitamin D was associated with a 49% lower risk of anxiety in physically active adults. To the best of our knowledge, no similar study has been published yet. Another cross-sectional study from Iran showed lower stress in women with better Vit D status (31). Although the following study was not done among athletes and did not measure dietary intake of vitamin D, it suggests the association between better vitamin D consumption and reduced risk of stress. In contrast, another study among colorectal cancer survivors found no significant link between serum vitamin D concentrations and the risk of anxiety (32). Due to the limited number of relevant studies, it is difficult to reach a firm conclusion about the association between vitamin D intake and the risk of anxiety among physically active adults. Earlier studies frequently found a significant association between vitamin D deficiency and the risk of anxiety (33). However, it is not clearly found that higher dietary vitamin D consumption might reduce anxiety scores. It is more important to be investigated among physically active persons, whose usual dietary intakes have been changed to reach better competition results.

All of the participants of this study were active and educated individuals and possibly affluent enough to choose their daily intake from high-quality foods.

Higher intake of vitamin D was linked to a lowered risk of sleep abnormalities in physically active adults in our study. Sleep quality is important for an athlete because sufficient relaxation improves physical abilities (34). We failed to find studies about the association of vitamin D intake with the risk of sleep disorders among physically active persons. An Iranian study showed that physical activity increased serum levels of vitamin D and through which reduced the prevalence of sleep disorders (35).

Vitamin D receptors are located in the different areas of the brain specifically in the substantia nigra, prefrontal cortex, hippocampus, thalamus, hypothalamus, and, cingulate gyrus which are considered responsible for the pathophysiology of depression. It has also been determined that the vitamin D activating enzyme 1-α-hydroxylase is highly distributed in different cell types of many brain regions, especially in neurons in the amygdala and glial cells in the hypothalamus. This distribution indicates a relationship between vitamin D and neuropsychiatric diseases such as depression, anxiety, and sleep disturbance (36). Antioxidant, anti-inflammatory, pro-neurogenic, and neuromodulator properties of Vit D are assumed to be important to making a human relax (37). Vit D deficiency has been associated with thinner cortex, reduced expression of genes related to cytoskeleton maintenance, synaptic plasticity, neurotransmission, cell proliferation, and growth, as well as a reduction in serum nerve growth factor (NGF) concentrations (38). Vit D interacts with its receptor (VDR) and then it allows other transcription factors to interact with this complex, through which it can affect gene expression (39). Some of these VDRs and vitamin D-metabolizing enzymes are found in the prefrontal cortex and hippocampus (40). In addition, stimulation of antioxidant enzymes by Vit D is known to contribute to redox homeostasis and anti-inflammatory processes (41, 42). Finally, vitamin D might change gut microbiota to improve mental health (43). Vitamin D deficiency is significantly prevalent in Iran. A meta-analysis by Tabrizi et al. (44) investigating vitamin D deficiency among Iranians estimated the prevalence of vitamin D deficiency among male, female, and pregnant women at 45.64, 61.90, and 60.45%, respectively. Vitamin D deficiency prevalence is considerably high in both males and females and it is also more prevalent in females, despite sufficient amounts of sunlight in all seasons in Iran and adequate exposure to sunlight according to some studies. There might be other reasons for this vitamin D deficiency. These might include the types of clothing in Iran, and other factors such as inadequate vitamin D intake, lifestyle, dietary habits, color, and air pollution (44).

To the best of our knowledge, this is the first study on the association of dietary vitamin D intake with the risk of depression, anxiety, and sleep disorders among physically active adults. However, the limitations of the current study should be also considered. Due to the cross-sectional design of the study, causality could not be conferred. Although we used validated questionnaires in our study, the probability of participants’ misclassification should not be neglected. We tried to select our participants from bodybuilding clubs to reduce heterogeneity between participants in terms of their exercise type, however, some differences remained. Furthermore, other mental health problems should also be studied in future investigations. Additionally, in our interviews, the daily exposure to the sun of participants was not asked from participants which should be investigated in future studies. This is a correlation study so there is no intervention or follow-up. Future studies should also investigate the correlation between serum levels of this vitamin and depression, anxiety, and sleep quality. Finally, we did not have an analysis on vitamin D in food intake of the subjects.

In conclusion, we found a significant association between dietary vitamin D intake and reduced risk of anxiety and sleep disorders, but not with the risk of depression, among physically active adults. Further observational studies among participants with different levels of physical activity are required to expand current knowledge in this area.

## Data availability statement

The datasets presented in this study can be found in online repositories. The names of the repository/repositories and accession number(s) can be found in the article/supplementary material.

## Ethics statement

The studies involving humans were approved by the Ethics Committee of Kashan University of Medical Sciences (IR.KAUMS.MEDNT.REC.1402.082). The studies were conducted in accordance with the local legislation and institutional requirements. Written informed consent for participation in this study was provided by the participants’ legal guardians/next of kin. Written informed consent was obtained from the individual(s), and minor(s)' legal guardian/next of kin, for the publication of any potentially identifiable images or data included in this article.

## Author contributions

VA: Methodology, Writing – original draft. MK: Formal analysis, Software, Writing – review & editing. MMi: Data curation, Investigation, Writing – review & editing. MMo: Writing – review & editing. AM: Supervision, Writing – original draft.
